# CRISPR/Cas9: Implications for Modeling and Therapy of Neurodegenerative Diseases

**DOI:** 10.3389/fnmol.2016.00030

**Published:** 2016-04-28

**Authors:** Weili Yang, Zhuchi Tu, Qiang Sun, Xiao-Jiang Li

**Affiliations:** ^1^Institute of Genetics and Developmental Biology, Chinese Academy of SciencesBeijing, China; ^2^Department of Human Genetics, Emory University School of MedicineAtlanta, GA, USA

**Keywords:** CRISPR/Cas9, neurodegenerative diseases, animal models

## Abstract

CRISPR/Cas9 is now used widely to genetically modify the genomes of various species. The ability of CRISPR/Cas9 to delete DNA sequences and correct DNA mutations opens up a new avenue to treat genetic diseases that are caused by DNA mutations. In this review, we describe the advantages of using CRISPR/Cas9 to engineer genomic DNAs in animal embryos, as well as in specific regions or cell types in the brain. We also discuss how to apply CRISPR/Cas9 to establish animal models of neurodegenerative diseases, such as Parkinson’s and Huntington’s disease (HD), and to treat these disorders that are caused by genetic mutations.

## The Development and Application of CRISPR/Cas9

CRISPR/Cas9, a recent addition to our tools for genome editing, has led to a revolution in biological research. CRISPR was originally reported as a set of short repeats located downstream of the iap gene in *E. coli* (Ishino et al., 1987). As more similar repeat elements were reported over years, Mojica et al. (2000) termed it as Short Regularly Spaced Repeats (SRSR). Jansen et al. (2002) then reported that several clusters of signature CRISPR-associated (Cas) genes were well conserved and typically adjacent to the repeat elements. Later, a series of studies uncovered the efficient antiviral defense mechanism of the CRISPR system (Jansen et al., 2002; Barrangou et al., 2007; Brouns et al., 2008; Karginov and Hannon, 2010). In this system, the non-coding CRISPR array is transcribed and cleaved within direct repeats into short crRNAs containing individual spacer sequences, which direct the nuclease Cas9 to targeted sequences of genomic DNA. The nuclease Cas9 then cuts both strands of DNA precisely, and the damaged DNA is repaired via non-homologous end joining (NHEJ) or homology-directed repair (HDR), thereby resulting in gene disruptions and inactivation of the targeted gene. Jinek et al. (2012) fused a crRNA containing the targeting guide sequence to a tracrRNA, called a single guide RNA (gRNA), to facilitate DNA cleavage by CRISPR/Cas9. CRISPR/Cas9 has now been used for genome editing in a variety of species (Hsu et al., 2014; Sander and Joung, 2014), especially in non-human primates that do not have embryonic stem cells for genomic manipulation (Niu et al., 2014; Chen et al., 2015) and human tripronuclear (3PN) zygotes (Liang et al., 2015).

Another powerful application of the CRISPR/Cas9 system is based on its ability to target many genomic loci simultaneously for studying gene function on a global scale (Koike-Yusa et al., 2014; Shalem et al., 2014; Zhou et al., 2014) which is certainly an advantage over RNAi and its limitations, such as low efficiency and specificity in genome-scale screens. Based on the DNA binding property of CRISPR/Cas9, researchers have also developed catalytically dead Cas9 (dCas9) to act as transcriptional or epigenetic regulators (Larson et al., 2013; Qi et al., 2013) or to couple Cas9 to fluorescence as DNA-binding reporters for live imaging (Chen et al., 2014).

The power of CRISPR/Cas9 to edit the genome holds great promise for treating human diseases caused by genetic DNA mutations. Recently, the UK Human Fertilization and Embryology Authority (HFEA) granted scientists in London permission to genetically edit human embryos within the range of research ethics (Callaway, 2016). Although there are still social and ethical issues that remain to be resolved, it is clear we must consider how to use CRISPR/Cas9 to treat human diseases in the future. In this review, we will focus on the application of CRISPR/Cas9 to animal models of neurodegenerative diseases.

## Use of CRISPR/Cas9 to Generate Animal Models of Neurodegenerative Diseases

Neurodegenerative diseases, such as Parkinson’s disease (PD) and Huntington’s disease (HD), share common features: namely, the age-dependent accumulation of misfolded proteins and selective neurodegeneration. For example, in PD, the presence of cytoplasmic misfolded proteins, termed Lewy bodies, which contain ubiquitinated alpha-synuclein, parkin, synphilin, and neurofilaments, are the pathological hallmark of this disease in patient brains. In the brains of HD patients, on the other hand, there are aggregates or inclusions formed in an age-dependent manner by mutant huntingtin with an expanded polyQ tract (Li and Li, 2011).

Animal models are highly valuable and have been used extensively to investigate neurological disorders and to find therapeutic targets for them. Because many neurodegenerative diseases can be caused by genetic DNA mutations, the ability of CRISPR/Cas9 to directly target any gene in one or two alleles of the embryonic genome opens up a new avenue for using this new technology to generate animal models of neurodegenerative diseases. The traditional gene targeting technology made it difficult to establish large animal models of human diseases due to the lack of embryonic stem cell lines. Since large animals are closer to humans, their disease models may more faithfully mimic the clinical symptoms of patients and are important for exploring the mechanisms and treatment of both neuropsychiatric disorders and age-related neurodegenerative diseases. For example, because the loss of function of the Parkin and Pink1 genes can cause PD, CRISPR/Cas9-mediated mutations can mimic knockout of the Parkin and/or Pink1 gene. CRISPR/Cas9 was found to functionally disrupt the dystrophin gene in founder monkeys and causes the same muscle atrophy phenotype seen in patients (Chen et al., 2015). Thus, when both alleles are mutated by CIRSPR/Cas9, the complete loss of Parkin or Pink1 will mimic the genetic mutations in PD patients. Also, because CRISPR/Cas9 can target multiple genes in the same cells, deletion of the Parkin and Pink1 genes will allow for studies of synergistic effects of loss of these important genes. Indeed, CRISPR/Cas9 has been used to generate pig models of PD by targeting the genes for Parkin, Pink1 and DJ1 (Zhou et al., 2015; Wang et al., 2016).

In addition to genome editing in germline cells, CRISPR/Cas9 can efficiently target genes in somatic tissues, such as neurons in the brain (Incontro et al., 2014; Platt et al., 2014; Straub et al., 2014; Swiech et al., 2015; Heidenreich and Zhang, 2016; Walters et al., 2016). In PD patients, a progressive loss of dopaminergic neurons in the substantia nigra is a key pathological feature. Thus, gRNAs and Cas9 can be delivered to the substantia nigra of animal brains by a viral system to investigate the effect of Parkin or Pink1 loss in adult brains. This approach is particularly useful for investigating the age-related neuropathology in PD.

Also, Cas9-mediated knock-in mutations within the genome can help generate animal models of those neurodegenerative diseases caused by a gain of toxicity of mutant proteins. For example, PD can be caused by mutations in α-synuclein, and HD is caused by polyQ expansion in huntingtin. Co-injection of Cas9/gRNAs with exogenous donor fragments carrying mutant sequences can replace the endogenous gene with mutant DNAs, thus creating animal models carrying the mutated sequences in the endogenous genes.

## CRISPR/Cas9-Mediated Treatment of Genetic Diseases

The animal models created by CRISPR/Cas9 normally carry mutations in endogenous genes and therefore provide better models to mimic human diseases than transgenic animals that express mutant genes under exogenous promoters. These new animal models will be highly valuable for identifying therapies using drugs or chemicals. Although CRSIPR/Cas9 has been used widely in the generation of a variety of cellular or animal models of human diseases, it is particularly important that we develop CRISPR/Cas9 as a therapeutic tool for treating human diseases. For example, CRISPR/Cas9 can be used to correct the causative gene mutations in monogenic recessive disorders or to inactivate the mutated allele in dominant-negative disorders to achieve therapeutic benefits. Recently, three groups independently reported that CRISPR/Cas9 was able to snip out a faulty exon of the dystrophin gene to generate a shortened but functional version of dystrophin to treat mice with muscular dystrophy (Long et al., 2016; Nelson et al., 2016; Tabebordbar et al., 2016). Although the mature muscle cells of adults lack the ability for cell division and have different DNA repair machinery than dividing cells, CRISPR/Cas9 gene editing can occur in skeletal muscle to functionally repair DNA mutations. In addition, CRISPR/Cas9 was used to correct the mutant Crygc gene that causes cataracts in zygotes from mice via HDR with an endogenous WT allele (Wu et al., 2013). All these findings suggest that CRISPR/Cas9 can modify the genome in any type of cell.

For neurodegenerative diseases, CRISPR/Cas9 can also be a powerful tool to eliminate the expression of mutant genes and therefore can alleviate the neuropathology caused by DNA mutations. For example, HD is caused by polyQ expansion in huntingtin, and selective targeting of the mutant huntingtin gene via CRISPR/Cas9 can be done in specific types of vulnerable neurons in the brain. Similarly, for transgenic PD animal models that express mutant α-synuclein, CRISPR/Cas9 can be designed to deplete the expression of mutant genes via NHEJ, which can lead to gene inactivation, in dopaminergic neurons. Furthermore, the ability of CRISPR/Cas9 to replace the mutant gene via HDR with normal DNA sequences can also lead to the genetic correction of DNA mutations in HD and PD animal models. Although the efficiency of such gene replacement is low at present, the rapidly developing CRISPR/Cas9 system offers a promising approach to generate knock-in models of human diseases.

## Challenges for CRISPR/Cas9

Despite the power of CRISPR/Cas9 for genome editing, there are still many challenges to be overcome when applying it to generate and treat animal models of human diseases. Because genome editing by CRISPR/Cas9 relies on approximately 23 base pair matches (Hsu et al., 2014), possible off-target effects have been considered an important issue. However, some studies reported that Cas9 could tolerate mismatches, depending on their distribution and number (Hsu et al., 2013; Mali et al., 2013; Fu et al., 2014). Also, a lower Cas9 concentration can decrease the off-target effect at the expense of on-target efficiency (Hsu et al., 2013). Thus, using specific gRNAs and appropriate Cas9 concentrations should minimize the off-targets and increase the specificity of CRSIPR/Cas9-mediated gene targeting.

The second issue with CRISPR/Cas9 is mosaic mutations, which may result from the prolonged expression of Cas9 after cell division or may be due to a slow rate of cleavage by Cas9 nuclease. Alternatively, differential DNA repair and non-homozygous recombination activities in zygotes and divided embryonic cells can also influence genetic mutation rates and mosaicism. Direct delivery of Cas9 protein into cells has also been tried and showed high target efficiency, but still resulted in mosaic mutations (Kim et al., 2014; Sung et al., 2014). Precise control of Cas9 nuclease expression at the transcriptional and translational levels in zygotes may reduce mosaic mutations.

Another challenge for CRISPR/Cas9 is the low rate of homologous recombination. Generally, HDR takes place in the synthesis (S) and the premitotic (G2) phases (Heyer et al., 2010), whereas NHEJ occurs in the growth 1 (G1) and the mitotic (M) phases (Daley and Sung, 2014). Although CRISPR/Cas9-mediated indel mutations via NHEJ have high efficiency, the HDR rate is relatively low. Suppression of NHEJ key molecules is found to increase the HDR rate by CRISPR/Cas9 (Chu et al., 2015; Maruyama et al., 2015). Further evolution of the CRISPR/Cas9 system to increase targeting specificity and efficiency is expected to improve the knock-in rate and the application of this genetic engineering tool to treat neurodegenerative diseases in the future.

## Author Contributions

WY, ZT, QS and X-JL wrote the review.

## Conflict of Interest Statement

The authors declare that the research was conducted in the absence of any commercial or financial relationships that could be construed as a potential conflict of interest.
